# Evaluating the Delivery of a Remote Osteoarthritis Peer Mentorship Programme for People Experiencing Socioeconomic Disadvantage

**DOI:** 10.1002/msc.70297

**Published:** 2026-11-06

**Authors:** Samantha Mason, Anna M. Anderson, Elizabeth C. Lavender, Linda Eckersley, Susan Barry, Mark Conner, Heidi Green, Gretl A. McHugh

**Affiliations:** ^1^ School of Healthcare University of Leeds Leeds UK; ^2^ Leeds Institute of Health Sciences University of Leeds Leeds UK; ^3^ Leeds Institute of Rheumatic and Musculoskeletal Medicine University of Leeds Leeds UK; ^4^ NIHR HealthTech Research Centre in Accelerated Surgical Care Leeds UK; ^5^ School of Psychology University of Leeds Leeds UK; ^6^ Freelance Aberdeenshire UK

**Keywords:** osteoarthritis, peer mentorship, remote delivery, self‐management, socioeconomic disadvantage

## Abstract

**Introduction:**

Hip and knee osteoarthritis can cause difficulty with daily activities and are particularly common in people experiencing socioeconomic disadvantage. Supported self‐management programmes, such as peer mentorship, can be a valuable way to support people to actively participate in managing their condition. The aim of this study was to develop, deliver and evaluate a remote peer mentorship programme to support self‐management of hip and/or knee osteoarthritis among people experiencing socioeconomic disadvantage.

**Methods:**

The current study is a process evaluation of a complex intervention involving participants with diagnosed osteoarthritis and self‐reported socioeconomic disadvantage. Prior to participating in the remote self‐management programme, mentees completed a baseline questionnaire. Following programme completion, mentees and peer mentors were invited to take part in a semi‐structured interview, with mentees also invited to a follow‐up interview. Quantitative data from baseline questionnaires were analysed using descriptive statistics, and an inductive‐deductive approach was undertaken to analyse qualitative data. Data were integrated through narrative using a contiguous approach.

**Results:**

Thirty mentees participated in the remote peer mentorship programme, delivered by 13 trained peer mentors. Twenty‐two mentees participated in an initial interview, 17 participated in a follow‐up interview, and 12 peer mentors were interviewed. Four inter‐related themes were developed that explored engagement, remote delivery, acceptability and self‐management progress. The process evaluation identified factors that influenced engagement with the programme such as competing priorities, the role of the peer mentor, and motivation.

**Conclusions:**

Remote peer mentorship for people with osteoarthritis experiencing socioeconomic disadvantage is largely acceptable, with the potential for meaningful impact.

## 1 Introduction

In the UK, approximately 5.4 million people have knee osteoarthritis and 3.2 million have hip osteoarthritis (OA) (Arthritis UK 2025). People with OA are more likely to have other long‐term conditions, such as cardiovascular disease, depression and diabetes (Swain et al. 2020; Hunter and Bierma‐Zeinstra 2019), which can increase disability and reduce quality of life (Dhalwani et al. 2016).

OA is more prevalent among those experiencing socioeconomic disadvantage (SED) (Reyes et al. 2015). SED refers to individuals living in less favourable social and economic conditions than the majority of society, and is typically characterised by factors such as low educational attainment, unemployment, and living in a deprived area (Gardner and Sherratt 2022). SED is disproportionately linked to being overweight or obese (Arthritis UK 2025), which increases the risk of developing knee OA (Reyes et al. 2015). SED is also linked to worse OA outcomes, such as increased OA severity, progression and reduced physical function (Luong et al. 2012). These factors exacerbate health inequalities, with disadvantaged groups experiencing poorer general health (Peoples et al. 2024), digital exclusion or barriers to accessing and using health services, such as longer waiting times and fewer opportunities to access care due to work, family commitments, or limited access to transport (Arthritis and Musculoskeletal Alliance 2024; Barakat and Konstantinidis 2023). People experiencing SED may have complex support needs and are typically underserved by research (Emery et al. 2023).

Self‐management programmes are a way to promote self‐care for people with chronic conditions and are designed to allow people to actively participate in the management of their condition (Almeida Silva et al. 2025; Foster et al. 2007). While peer support is recommended to enable self‐management (Arthritis and Musculoskeletal Alliance 2024), there is a lack of strong supporting evidence for the effectiveness of such programmes for adults living with chronic musculoskeletal pain (Wilson et al. 2024).

The current study builds on our previous work, which assessed the feasibility of an in‐person peer mentorship programme, designed to support people to self‐manage their hip and/or knee OA (Anderson et al. 2021). This work identified understanding, confidence, and motivation as key enablers for improved self‐management behaviours. The peer mentor‐mentee relationship was imperative to the programme’s success, with personalised support and bonding over shared experiences acting as facilitators to engagement (Lavender et al. 2022a, 2022b).

This study aimed to develop, deliver, and evaluate a remote peer mentorship programme to support self‐management of hip and knee OA among people experiencing SED, and assess suitability, acceptability, and perceived usefulness. We adapted our in‐person programme to a remote format to reflect the increased use of remote healthcare delivery catalysed by the COVID‐19 pandemic, reduce logistical barriers, and enhance reach, flexibility and convenience. We developed the programme with, and for, people experiencing SED to address a research and service provision gap, and to provide evidence for a peer mentorship programme for hip and knee OA that contributes to reducing health inequalities.

## 2 Materials and Methods

### 2.1 Study Design

The current study is a process evaluation of a complex intervention, following Medical Research Council (MRC) guidelines (Moore et al. 2015a; Moore et al. 2015b). The study’s reporting is guided by the Consolidated Criteria for Reporting Qualitative Research (COREQ) checklist (Tong et al. 2007). To enhance transparency, the completed COREQ checklist is provided in Supporting Information S1: Table 1.

### 2.2 Participants and Recruitment

A wide recruitment strategy was used to reach adults living with hip and/or knee OA and experiencing SED. This included: targeted text messaging through a network of GP practices serving areas with high levels of SED, based on IMD (Ministry of Housing 2019); use of a community engagement agency specialising in improving access to health research for underserved populations; and promotion via social media, local and national third sector organisations and local community healthcare NHS trust musculoskeletal services.

Individuals were eligible for inclusion if they were at least 18 years old, had a diagnosis of hip and/or knee OA and self‐identified as experiencing SED. SED was assessed during screening by asking potential participants if they were experiencing SED. If this question was not understood, the question was re‐phrased to ask whether they felt they had missed out on opportunities due to their financial, educational or social circumstances. The decision to enable participants to self‐identify as experiencing SED was based on views expressed by public contributors in a previous project, who highlighted the dynamic nature of socioeconomic status (Sherratt et al. 2026).

Recruitment of participants took place from July 2023 to April 2024. We received expressions of interest from 100 potential participants. Of these, 42 were eligible to take part and 34 consented. Thirty‐two participants were matched with a volunteer peer mentor, and 30 participants started the remote peer mentorship programme. Four people withdrew after consenting. Full details of participant recruitment are presented in Figure 1.

**FIGURE 1:**
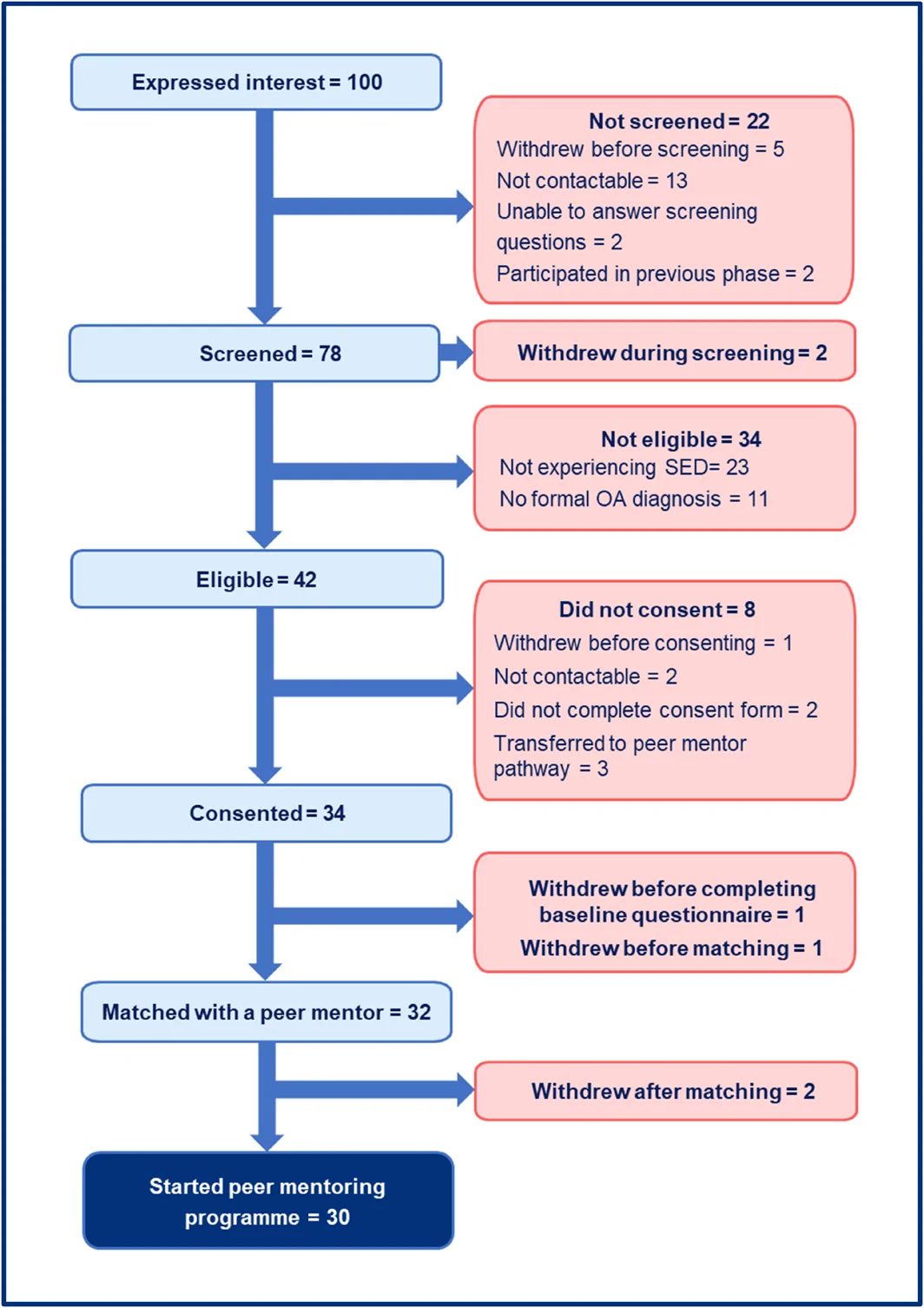
Recruitment of mentees.

Recruitment of volunteer peer mentors took place from August 2023 to January 2024. Volunteers were recruited through national (VOICE, Arthritis UK) and local community agencies (Leeds Older People’s Forum, Health For All) based in more deprived areas, via posters, newsletters and in person, via social media posts targeting people with OA and via ethnically diverse groups. Of the 35 people who expressed interest in becoming a volunteer peer mentor, 23 were eligible. After five people withdrew and one person elected to be a mentee instead, 17 people were invited to attend the training. Fourteen people attended peer mentor training and 13 became peer mentors. Further details of the training and recruitment of peer mentors are reported elsewhere (Lavender et al. 2025).

### 2.3 Intervention

The intervention was previously delivered as an in‐person peer mentorship programme (Anderson et al. 2021), and adapted to be delivered remotely to people experiencing SED. Adaptations to address barriers related to digital confidence, literacy and access included; changes to content, supporting resources, intervention delivery, mentorship sessions and delivery of peer mentor training (Anderson et al. 2025; Lavender et al. 2025).

Trained volunteer peer mentors delivered the adapted remote peer mentorship programme to mentees. Each mentee was matched with a peer mentor and invited to attend six remote peer mentorship sessions via videoconferencing or phone calls. Sessions provided mentees with knowledge, skills and support to self‐manage their hip and/or knee OA. Mentees were asked if they would prefer one‐to‐one sessions with their peer mentor or sessions comprising two mentees and one peer mentor. Peer mentorship sessions were designed to be tailored to the needs of each mentee. Peer mentors were advised to cover all core topics and then be led by the mentees about which optional topics to cover. To support sessions, mentees were sent programme resources by post and electronically. Details of peer mentoring sessions are presented in Supporting Information S1: Table 2 and a summary of the process for programme delivery is presented in Figure 2.

**FIGURE 2:**
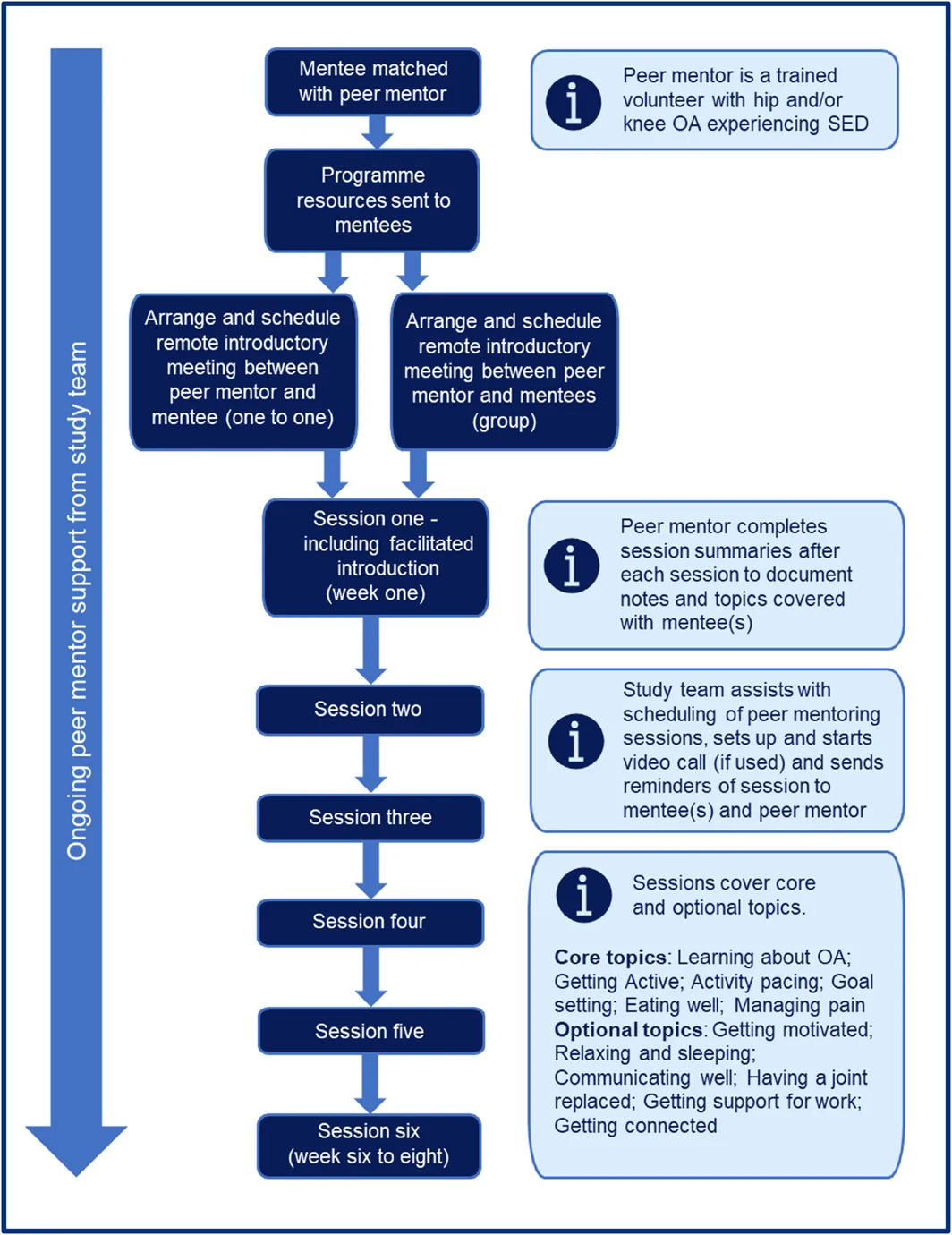
Process for programme delivery.

### 2.4 Data Collection

#### 2.4.1 Quantitative Baseline Survey

Before participating in the intervention, mentee baseline data was collected via questionnaires. Questionnaire content included:

- Participant characteristics/demographics
- History of arthritis
- Belief in their ability to manage their OA (8‐item Arthritis Self‐Efficacy Scale) (Lorig et al. 1989)
- Health‐related quality of life (EQ‐5D‐5L) (EuroQol Group 1990)
- Pain ratings
- Use of pain management
- Perception and adequacy of social support
- Exercise
- Confidence in using digital technology.

#### 2.4.2 Session Summaries

Session summaries of each mentorship session were completed by peer mentors. Summaries provided information about programme delivery, how mentees interacted with the programme, and perceived behaviour change.

#### 2.4.3 Qualitative Interviews

Mentees were invited to take part in remote longitudinal qualitative semi‐structured interviews: an initial interview after they completed the remote peer mentorship programme at approximately 6 weeks; and a follow‐up interview approximately 6 months after beginning the programme. Peer mentors were invited to take part in a remote semi‐structured interview upon completion of peer mentoring.

Topic guides for mentee interviews were based on the constructs of Normalisation Process Theory (May et al. 2009; Murray et al. 2010) and the Framework of Implementation Outcomes (Proctor et al. 2011). The initial mentee interview explored topics including:

- experiences of remote OA peer mentorship;
- health‐related changes or decisions made because of the programme;
- benefits and challenges of peer mentorship.

Follow‐up interviews explored sustained self‐management including:

- perceptions of self‐management changes;
- changes in general health and OA symptoms;
- knowledge of self‐management;
- confidence to self‐manage their OA;
- perceived usefulness of the remote peer mentorship programme.

Initial interviews with mentees took place between May and September 2024 and lasted between 23 and 58 minutes (mean: 42 minutes). Follow‐up interviews took place between September and December 2024 and lasted between 25 and 63 minutes (mean: 40 minutes). All interviews were conducted by EL or SM.

Peer mentor interviews explored their experiences of receiving training and support, being a peer mentor, and delivering the programme. Peer mentors were interviewed by SM between July and September 2024. Interviews lasted between 41 and 109 minutes (mean: 70 minutes).

Interviews were digitally recorded using an encrypted audio‐recorder and/or the record function of Microsoft Teams/Zoom. Researchers kept written reflections of the interviews. Recordings were transcribed verbatim by a professional transcription company or Microsoft Teams/Zoom. Transcripts were verified for accuracy and pseudonymised. Personally identifiable information was removed to maintain participant confidentiality.

#### 2.4.4 Data Analysis

Quantitative data from baseline questionnaires were analysed using descriptive statistics. Session summaries were analysed using content analysis.

An inductive‐deductive approach was taken for qualitative data analysis. Reflexive thematic analysis was conducted to develop themes and fully explore and interpret qualitative interview data, initially using an inductive approach (Braun and Clarke 2006; Braun 2021). Analysis was primarily undertaken by two female researchers with qualitative research experience (SM, EL). Both researchers read the transcripts for familiarisation. Patterns within the data were recorded, initial codes were inductively generated, and the data were organised into meaningful groups. Microsoft Excel was used to organise data. Inductive coding was undertaken to identify themes that reflected the key aspects of the intervention, its objectives, and components that may have impacted engagement. During discussion between the two researchers (SM, EL), pseudonyms for participants were agreed and themes were reviewed, refined, defined and labelled. While some themes were identified at a semantic (descriptive) level, others were developed through a more interpretative process based on what participants considered important or meaningful about the programme. Findings were reviewed with other project team members. An analytic narrative was written for each theme and supported by data extracts.

Deductive analysis was undertaken using the MRC process evaluation framework (Moore et al. 2015b). Themes were re‐evaluated, re‐labelled and assigned to the inter‐related MRC process evaluation framework components:

- Context—anything external to the intervention that influences implementation, effectiveness or outcomes.
- Mechanisms of impact—explain how the delivered intervention produces change and how these interactions result in outcomes.
- Mediators—represent specific, measurable elements within the broader mechanisms of impact and describe the intermediate processes that explain outcomes.

Fidelity was assessed using the session summaries and qualitative data, to determine whether the intervention was delivered as intended, and evaluate the effect of deviations.

The process evaluation utilised qualitative data to explore how mentees interacted with the intervention and understand how contextual factors influenced programme delivery and engagement. Baseline quantitative data from mentees were used to provide demographic information and to inform understanding of contextual factors.

Mentee data was complemented by peer mentor qualitative data and findings from the session summaries. Quantitative and qualitative data were initially analysed separately and then integrated through narrative at the interpretation and reporting stage using a contiguous approach (Fetters et al. 2013).

### 2.5 Ethical Approval

Ethical approval for this study was obtained from the South Birmingham Research Ethics Committee (23/WM/0108). All participants completed an electronic consent form, providing their consent to take part in the programme and to be interviewed. Participants who took part in follow‐up interviews completed an additional electronic consent form. Participants also gave verbal consent at the beginning of the interview for their interview to be recorded and were assured that transcripts would be anonymised.

## 3 Results

### 3.1 Participants: Peer Mentors

Peer mentors consisted of three males and ten females, aged between 32 and 72 years, typically living with either bilateral knee OA (*n* = 4) or bilateral hip OA (*n* = 4). Further details about the peer mentors are reported elsewhere (Lavender et al. 2025). Following completion of the programme, 12 peer mentors were interviewed.

### 3.2 Participants: Mentees

Thirty mentees aged between 34 and 82 years began the remote peer mentorship programme. Nine were male (30%) and 21 were female (70%). Detailed information about the mentees is provided in Table 1.

**TABLE 1:** Characteristics of mentees.

Characteristic	*N*	%
Sex		
Male	9	30.00%
Female	21	70.00%
Age range		
30–39 years	1	3.33%
40–49 years	4	13.33%
50–59 years	7	23.33%
60–69 years	10	33.33%
70–79 years	7	23.33%
80+ years	1	3.33%
Ethnicity		
White	20	66.67%
Black‐African	2	6.67%
Indian	6	20.00%
Pakistani	2	6.67%
Geographical region		
Brighton and Hove	1	3.33%
Greater London	1	3.33%
Leicestershire	5	16.67%
South Yorkshire	8	26.67%
West Yorkshire	15	50.00%
Site of OA (hip and knee only)		
Single knee	6	20.00%
Single knee and single hip	4	13.33%
Single knee and both hips	6	20.00%
Both knees	9	30.00%
Both hips	2	6.67%
Both knees and hips	3	10.00%
Length of time with OA		
1 year	2	6.67%
Between 2 and 5 years	10	33.33%
Between 6 and 10 years	9	30.00%
Between 11 and 15 years	2	6.67%
Over 15 years	7	23.33%
Digital capability		
Unconfident	7	23.33%
Neither confident nor unconfident	5	16.67%
Confident	11	36.67%
Very confident	7	23.33%

Baseline questionnaires indicated that all mentees experienced pain from their OA, with 80% (*n* = 24) reporting that their pain was often or always present. On a scale of 0–10, 73% (22/30) reported a pain score of ≥ 6 (mean: 6.7, SD = 2). Mentees reported a mean eight‐item arthritis self‐efficacy scale score of 4.3 (SD = 1.8), with scores ranging from 1.6 to 7.6, indicating that none of the mentees felt certain that they could perform specific tasks without their OA having an impact. Ratings on the EQ‐5D‐5L indicated that 80% of the mentees had moderate or severe mobility issues. Data from the baseline questionnaire are presented in Table 2.

**TABLE 2:** Mentee baseline data.

Measure	Question/statement	*N* (%)	Mean	SD	Min	Max
History of arthritis	Currently suffer with pain because of your arthritis	30 (100.00)				
	My pain is sometimes present	6 (20.00)				
	My pain is often present	6 (20.00)				
	My pain is always present	18 (60.00)				
	How would you rate your current level of pain? At one end no pain [0] at the other end extreme pain [10]	30 (100.00	6.67	1.98	2	10
Current exercise frequency	Daily	6 (20.00)				
	4–6 times per week	3 (10.00)				
	2–3 times per week	8 (26.67)				
	Once per week	4 (13.33)				
	Less than once per week	2 (6.67)				
	Rarely/Never	7 (23.33)				
EQ‐5D‐5L Mobility	I have no problems walking about	0 (0.00)				
	I have slight problems in walking about	6 (20.00)				
	I have moderate problems in walking about	17 (56.67)				
	I have severe problems in walking about	7 (23.33)				
	Unable to walk about	0 (0.00)				
EQ‐5D‐5L Self‐care	I have no problems washing or dressing myself	5 (16.67)				
	I have slight problems washing or dressing myself	8 (26.67)				
	I have moderate problems washing or dressing myself	13 (43.33)				
	I have severe problems washing or dressing myself	4 (13.33)				
	I am unable to wash or dress myself	0 (0.00)				
EQ‐5D‐5L Usual activities	I have no problems doing my usual activities	2 (6.67)				
	I have slight problems doing my usual activities	8 (26.67)				
	I have moderate problems doing my usual activities	15 (50.00)				
	I have severe problems doing my usual activities	3 (10.00)				
	I am unable to do my usual activities	2 (6.67)				
EQ‐5D‐5L Pain/discomfort	I have no pain or discomfort	0 (0.00)				
	I have slight pain or discomfort	7 (23.33)				
	I have moderate pain or discomfort	14 (46.67)				
	I have severe pain or discomfort	7 (23.33)				
	I have extreme pain or discomfort	2 (6.67)				
EQ‐5D‐5L Anxiety/depression	I am not anxious or depressed	12 (40.00)				
	I am slightly anxious or depressed	6 (20.00)				
	I am moderately anxious or depressed	8 (26.67)				
	I am severely anxious or depressed	3 (10.00)				
	I am extremely anxious or depressed	1 (3.33)				
EQ‐5D‐5L Visual analog scale	How good or bad your health is TODAY on a scale from 0–100	30 (100.00)	49.33	19.27	10	85
8‐Item arthritis self‐efficacy scale	How certain are you that you can decrease your pain quite a bit?	30 (100.00)	4.20	2.20	1	8
	How certain are you that you can keep your arthritis pain from interfering with your sleep?	30 (100.00)	3.60	2.01	1	7
	How certain are you that you can keep your arthritis pain from interfering with the things you want to do?	30 (100.00)	3.90	1.90	1	7
	How certain are you that you can regulate your activity so as to be active without aggravating your arthritis?	30 (100.00)	4.33	2.09	1	8
	How certain are you that you can keep the fatigue caused by your arthritis from interfering with the things you want to do?	30 (100.00)	4.17	2.28	1	9
	How certain are you that you can do something to help yourself feel better if you are feeling blue?	30 (100.00)	5.23	2.22	1	10
	Compared with other people with arthritis like yours, how certain are you that you can manage pain during your daily activities?	30 (100.00)	4.70	2.20	1	8
	How certain are you that you can deal with the frustration of arthritis?	30 (100.00)	4.43	2.60	1	10

Thirteen mentees completed all six remote peer mentorship sessions (43%), four completed five sessions (13%), one mentee completed four sessions (3%), five completed three sessions (17%) and seven mentees completed fewer than three sessions (23%). Reasons for non‐completion of sessions are detailed in Supporting Information S1: Table 3.

Following completion of the programme, 20 mentees participated in remote interviews, with two related mentees participating in a remote interview together. Seventeen mentees took part in a follow‐up interview.

### 3.3 Themes

#### 3.3.1 Theme One: Mentee Engagement With the Peer Mentorship Programme

Engagement with the peer mentorship programme was strongest when mentees felt that they had developed rapport and trust with their peer mentor. Most mentees reported a good relationship, describing their peer mentor as positive, approachable and compassionate. Some mentees reported that the peer mentor felt like a friend, which mostly facilitated engagement. However, for a few mentees, this friendship became a distraction from the purpose of the programme.

Peer mentors’ lived experience of hip and/or knee OA and understanding the associated daily challenges increased their credibility and relatability with mentees, which enhanced engagement. Several mentees reported how consistent, dedicated and unhurried time with their peer mentor was more helpful than previous OA‐related healthcare referrals.

I’ve just got more from Isla with being on the phone and having a session with her than I have with going to a physiotherapist.

(Cheryl, mentee, initial interview)

Engagement improved when self‐management guidance was tailored to the mentee’s daily routine and emphasised actionable strategies, including gradually increasing activity and adapting challenging tasks.

#### 3.3.2 Theme Two: Remote Delivery of the Peer Mentorship Programme

Some mentees only considered participating because of the remote delivery of the peer mentorship programme, describing the format as more convenient and better aligned with their current routines.

Remote delivery made it more challenging for some mentees to build relationships with their peer mentors. Technical issues during videoconferencing contributed to further disengagement of a mentee that was already minimally engaged with the programme.

Peer mentors were more likely to report difficulties or anxiety around online connectivity when using videoconferencing platforms. Some peer mentors experienced challenges with remotely demonstrating or practising programme exercises with their mentees, citing limited space where they could demonstrate the exercises and be easily visible to their mentees via videoconferencing, or safety concerns associated with practising exercises over the phone.

We ended up on the WhatsApp and I just, it didn’t feel safe to me. I didn’t think first that I should be doing any sort of exercise with it, with somebody I couldn’t see, because I couldn’t see what she was doing.

(Isla, peer mentor)

#### 3.3.3 Theme Three: Acceptability of the Peer Mentorship Programme

Most mentees reported gaining something positive from the peer mentorship programme, such as more knowledge of self‐management strategies, or improved confidence to engage with self‐management. Many described being more motivated or better equipped to deal with their condition.

The programme resources were considered helpful, especially the programme exercises and culturally specific ‘Eatwell guide’. Mentees liked having the resources to refer to after the programme had finished, with some mentees regularly referring to the resources at follow‐up.

Programme delivery by a peer mentor was particularly valued. Mentees appreciated the combination of emotional and practical support.

It was good speaking to someone who understood how you felt or how I felt, and not only that. The emotional part of it, but also the like, the physical aspects of it, the problems trying to get medication and things sorted out, dealing with doctors and hospitals and things. It was a full package.

(Denise, mentee, initial interview)

#### 3.3.4 Theme Four: Self‐Management Progress

Most mentees reported progress with implementation of self‐management. Initial interviews indicated that mentees were trying a variety of self‐management strategies, with programme exercises, activity, and eating well being the most utilised forms of self‐management. At follow‐up, mentees understood which self‐management strategies worked for them.

Understanding the purpose of self‐management and how to implement self‐management in daily life was key to progress. Activity pacing was the self‐management strategy that was most variably understood by mentees. In initial interviews, some mentees understood that they could modify activity when they experienced OA pain, while others interpreted activity pacing as restricting activity. Session summaries indicated that activity pacing was not consistently covered, which may have contributed to understanding. At follow‐up, understanding had improved, but some mentees only understood the concept after overdoing activity and experiencing increased pain or fatigue, leading to resting and reducing activity (‘boom and bust’). Other mentees perceived activity pacing as unhelpful for tasks that they believed required completion within a specific time frame.

In follow‐up interviews, mentees discussed progression since joining the peer mentorship programme. This included building self‐management into daily life, forming consistent habits, recognising capability improvements (e.g., walking further), improved OA symptoms (e.g., decreased pain, improved mobility) and feeling more able to cope with their OA.

The person I am now is completely different to the person I was at both at the beginning and at six weeks. I think at six weeks I was sort of, I was sitting and thinking of things… the mentorship has sort of opened my eyes more.

(Denise, mentee, follow‐up interview)

### 3.4 Structuring Findings Using the MRC Process Evaluation Guidelines

Deductive analysis utilised findings from mentees’ and peer mentors’ qualitative data, content analysis from the session summaries (27) completed by peer mentors, and quantitative findings from mentees’ baseline questionnaires (30). The findings illustrate how the intervention functions in both the short term (Figure 3) and long term (Figure 4).

**FIGURE 3:**
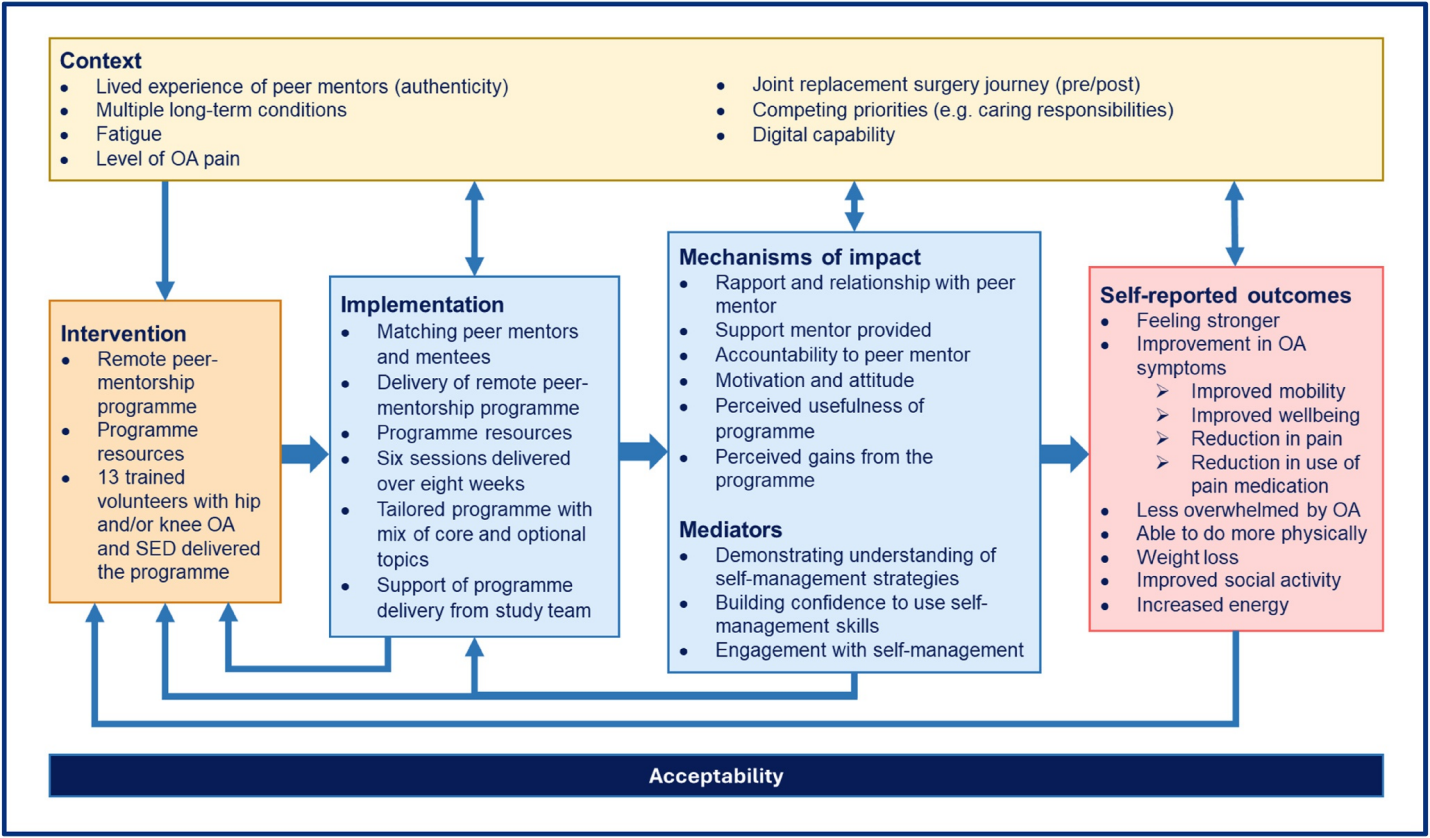
Process evaluation diagram illustrating how the intervention functions in the short term.

**FIGURE 4:**
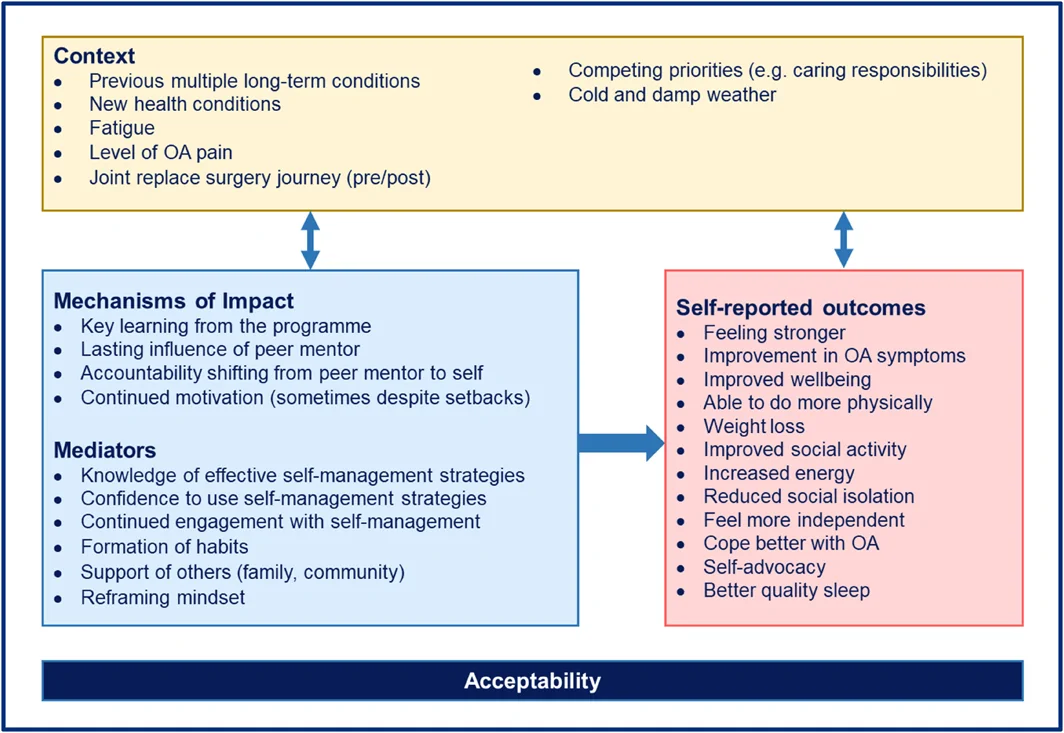
Process evaluation diagram illustrating how the intervention functions in the long term.

### 3.5 Implementation

The main challenges to implementation of the remote peer mentorship programme are presented below. The intervention process is presented in Figure 2 and all elements of implementation are included within Figure 3.

#### 3.5.1 Delivery of the Remote Peer Mentorship Programme

Programme delivery was inconsistent. Evidence from session summaries showed that activity pacing and pain management were not consistently covered by peer mentors. Peer mentors were encouraged to tailor the peer mentorship programme to the needs of their mentees. For some, tailoring meant prioritising topics that were relevant for their mentees (e.g., getting active, eating well, and goal setting). Some peer mentors felt that fidelity varied, as they spent longer than anticipated building trust and rapport with mentees, or offered predominantly emotional support without adequately balancing this with practical guidance.

It was very, very hard. I’d say the first three weeks, and by week three I felt like giving up. And then suddenly he changed. Suddenly it was like, maybe it was the trust that I’d built by coming, you know, calling him same time each week.

(Nora, peer mentor)

Balancing personal conversations and programme delivery was challenging for several peer mentors. One peer mentor expressed having to spend more time than she considered appropriate ensuring that her group of two mentees remained focussed on session content.

I felt like I wasn’t doing the study justice. I felt like I wasn’t doing it properly and I hate not doing things properly. Yeah, I felt like sometimes they weren’t listening because they’d have little side chats as well.

(Suzy, peer mentor)

A further challenge was that several mentees did not want to practise programme exercises with their peer mentors. Some mentees explained that they were familiar with the exercises through previous physiotherapy, but others did not provide a reason.

#### 3.5.2 Support From the Study Team

To prevent digital exclusion and optimise inclusion of participants who were not digitally confident, internet‐enabled tablets were offered to all study participants along with ongoing digital support and training from the study team and through a Patient and Public Involvement (PPI) digital champion. The option to participate via phone was also offered.

The research team provided ad hoc practical guidance and emotional support to both mentees and peer mentors. Two co‐applicants/PPI members offered additional support to peer mentors, if needed, by advising on session structure and co‐delivering several peer mentorship sessions to help build confidence.

### 3.6 Context

Contextual factors affected how mentees engaged with the peer mentorship programme. All contextual factors are presented in Table 3.

**TABLE 3:** Intervention contextual factors.

Context	Description	Exemplar quotes
Lived experience of peer mentors	Peer mentor having lived experience of hip and/or knee OA was considered valuable by mentees as they perceived the mentor to be relatable, similar to themselves and authentic.	‘The best thing I got out of this was speaking to Lisa because she knows what it’s like. She knows the challenges you face. It’s not just someone who’s got the medical knowledge. She’s got the personal knowledge which I think is 100% better than speaking to someone who’s just got the textbook’ *Janice (initial interview)*
		‘I thought it was very useful. It was good speaking to someone who understood how you felt or how I felt’ *Denise (initial interview)*
Multiple long‐term conditions	27/30 mentees reported other long‐term conditions in addition to their hip/knee OA. The severity of these other long‐term conditions affected how well some mentees engaged with peer‐mentorship and self‐management.	‘I think it, it is the nerve pain which is causing this problem, which I’m still suffering. I can’t walk properly, and I am suffering with the stick. My foot is very cold and feels, you know, very, very cold all the time, and it gets swollen as well if I’m walking too much’ *Kita (initial interview)*
		‘It’s very difficult when you’ve got so many different things wrong. And the primary thing for me is that, you know, I keep breathing’ *Sarra (follow‐up interview)*
Fatigue and level of OA pain	Fatigue, energy and OA pain were all cited by mentees as barriers to being able to engage with self‐management, especially eating better, activity and programme exercises.	‘I do suffer with fatigue quite a lot… It will be like one day I could have the energy all day and then for the next two days, just wiped out and that’s the way it goes… it’s just learning to go up and down isn’t it?’ *Marie (initial interview)*
		‘We have arthritis. Even if I want to go for a longer walk or do more walking, you can’t because you’re in pain, you can’t do a lot of exercise’ *Ruby (initial interview)*
Joint replacement surgery journey	Some mentees were awaiting joint replacement surgery at the time of the peer‐mentorship programme. These mentees had differing levels of engagement with the programme compared to those who had recovered or were recovering from joint replacement surgery (even if the joint replacement was perceived as unsuccessful). This was usually linked to fatigue and level of OA pain.	‘Three of the Fridays she rang me, I was in so much agony with my knees. Right, I just couldn’t… I couldn’t do any exercises or anything’ *Ernie (initial interview)*
		‘When I went for my operation for my knee, everything was fine. Everything seems to have gone wrong since then’ *Fred (initial interview)*
Competing priorities	Competing priorities included caring for loved ones, work, hobbies and managing other health conditions. This meant that self‐management was not always a priority, and other things or the needs of others often took priority.	‘At first I felt bad because I was trying to talk to Suzy and obviously I’ve got my daughter and I said, I explained, I was like, I don’t think I’m gonna be able to carry on, because, like I didn’t want her to think I was being rude, with my daughter interrupting but she were happy. She were like, no, it’s all right. You can just listen in, or you can have your camera off, or any of that. So I was like, oh, that’s all right then’ *Gemma (initial interview)*
		‘It would have been better if I’d have made it earlier from the start… I mean, I can’t always predict what’s gonna happen. I can’t plan too far ahead apart from appointments, doctors, appointments, hospital appointments or holidays or whatever I mean, apart from things that are fixed well in advance, I can’t always say what I’m going to be doing day to day’ *Brian (initial interview)*
Digital capability	Attendance or length of peer mentoring sessions was variable due to issues connecting online or using videoconferencing. This may have contributed to some mentees dropping out of the programme. Other mentees were reluctant to use videoconferencing for remote peer mentorship, preferring phone calls.	‘I don’t do video conferences on the phone because, let’s put it this way, I’m a bit sort of scared of other people may be able to tap in, if you know what I mean. So telephone conversation was much better for me anyway’ *Ernie (initial interview)*
		‘I think it was a bit disjointed because of the fact that I think, because I couldn’t get it on the desktop and I was on the phone. The fact that I didn’t have the camera, both ways, you know, face to face. When it gets down to being more like a telephone conversation. It’s not, it’s not quite the same’ *Brian (initial interview)*
Cold and damp weather	For some mentees, self‐managing their OA symptoms was more challenging during winter months or their OA symptoms were more severe.	‘I mean they can be six and then sometimes they’re ten at the moment and especially now, with the cold weather coming… when the cold started, the weather changed, I was wearing light clothes and then the cold goes into my bones quick! you know what I mean? Then that slows you down again’ *Samira on pain levels out of 10 (follow‐up interview)*
		‘of course this cold weather is not helping it much either’ *Ernie (follow‐up interview)*

#### 3.6.1 Fatigue and Level of OA Pain

Fatigue and OA pain were barriers to engaging with the programme and significantly affected quality of life. Some mentees were unable to consistently practise self‐management due to variable pain or energy levels, while others were limited to a small number of programme exercises, often focussing on lower impact options.

#### 3.6.2 Competing Priorities

Caring responsibilities most frequently affected engagement and attendance. While some mentees described the challenges of balancing life, caring responsibilities and self‐management, others valued the programme as personal time that supported self‐care and prioritised their well‐being. Data from follow‐up interviews indicated that some mentees were prioritising themselves more or including the people they cared for within self‐management (e.g., going for walks together). Other competing priorities included work, hobbies and managing other health conditions.

### 3.7 Mechanisms of Impact

Motivation and attitude had the greatest influence on programme engagement. All mechanisms of impact are presented in Table 4.

**TABLE 4:** Intervention mechanisms of impact.

Mechanism of impact	Description	Exemplar quotes
Rapport and relationship with the peer mentor	Feeling well connected and comfortable with the peer mentor and perceiving that the peer mentor was interested led to increased engagement with the programme, while feeling dismissed by the mentor was a barrier to engaging.	‘Nora was absolutely fantastic. We got on like a house on fire from the very first phone call. She made me feel very comfortable’ *Ernie (initial interview)*
		‘I just felt like I was there as an inconvenience in some way. Maybe that’s my perception. But then it was like, oh, well, I’ve got this job and I won’t be able to this day, that day, the other day, this time, that time and I was like thinking What? Like I thought we’d arranged that we’re going to do it on this day… because I have a life as well’ *Sarra (initial interview)*
Support peer mentor offered	Mentees received both emotional support such as encouragement, reassurance, and providing a boost when things were difficult for the mentee; and the practical support, including sharing experiences and advice, answering questions, and tailoring programme content.	‘My mentor was my sounding board especially for the first couple of sessions’ *Denise (initial interview)*
		‘Talking through situations with other people like Richard, and it’s like, “well, have you thought about this”? “No, I haven’t, never”. And then it’s like, so me in the past I’d have gone,” yeah, I’m not doing that”. But actually sat and thought about it. I Thought if I really want to do something and something’s stopping me from doing it this way, why don’t I try that way? And I’ve done it and I’ve tried it and I’m like actually he is speaking sense here’ *Gail (initial interview)*
Lasting influence of peer mentor	Mentees discussed what the peer mentor provided during the peer‐mentorship programme that was valuable and memorable to the mentee in the long term.	‘I really looked forward to Saturday mornings to be honest with you, because I knew it was going to be my hour to talk to somebody that’s in a similar situation and it’s just. It’s just, you learn so much from it. It’s only an hour, I know, and it was only whatever 6 weeks. But I learnt so much from Victoria and I hope that she learnt little bits from me, too’ *Heather (follow‐up interview)*
		‘Isla called every week. She was, there was no rush. There was nothing in a rush. She had plenty of time to speak, she was really nice and then she always followed back up’ *Cheryl (follow‐up interview)*
Accountability	Mentees discussed feeling a sense of responsibility to not let their peer mentor down during their initial interviews. At follow‐up, some mentees were not able to keep up with self‐management without their peer mentor to be accountable to.	‘At least I’m doing them. Whereas in the past, I wouldn’t… If I wasn’t doing them and having to admit to Daphne when she’s putting her time in, I’d feel as if I was letting her down’ *Sheila (initial interview)*
		‘When I was with Lisa we had like little goals that she gave me each week, and I kept to the goals, but since I haven’t had her to nag me each week. I must admit I haven’t done all the things that I had planned on doing in so far as exercise’ *Janice (follow‐up interview)*
Motivation and attitude	Motivations to self‐manage and their attitude towards self‐management and specific topics, how the programme impacted on motivation and whether motivation was sustained.	‘This is to get better so I can get about a bit more and then another thing, the older you’re getting, the worse it will be getting, won’t be getting any better. But if you don’t manage it now, then it is, it’ll be like then it was too late’ *Samira (initial interview)*
		‘I’ve got more energy, I feel better in myself just for exercising. Tell you truth, because I obviously can’t get to the gym, so I feel a lot better, and I feel like I can do a little bit more on a daily basis and just the overall of getting up and getting motivated, because it’s easier just to stay in and sit down and not move out of a chair. So just to have the motivation to get up, do my exercises, do them every day, stay consistent. It does make you feel a lot better in yourself’ *Cheryl (follow‐up interview)*
Perceived gains from the programme	The main gains described were knowledge of OA, knowledge of self‐management strategies and confidence to use self‐management strategies.	‘She was saying how to pace myself and that really did help me a lot and not to go in it like a bull in a china shop and also with certain foods that you can eat that is better for you than other foods’ *Diane (initial interview)*
		‘She gave me more confidence, you know, she says, you know, just go out for walking. Do the slow walking in the in the garden. Just walk a little bit in the house. When it is winter you can walk in the house, then you can slowly, gradually, you know, if you’re properly able to’ *Kita (initial interview)*
Perceived usefulness of the programme (the degree that people perceive the programme to be useful to them)	Discussions focussed on why mentees found the programme useful and what aspects of the programme or self‐management strategies were considered to be the most important.	‘Main benefits was like the mindfulness and, oh we’ve got a session today and like catching up on our experiences and going through the book’ *Gemma (initial interview)*
		‘I’ve never spoke to anybody apart from the Doctor, whose got hundreds of patients to see a day and they just like on a production line. He, Mark was genuinely, you know, thinking about what he was saying to me’ *Fred (initial interview)*
Key learning	Key learning that mentees took away from the programme, especially long‐term.	‘The research has helped me to really focus on what I want out of life, put things into place, pace myself, understand myself and know more about medication stuff’ *Chika (follow‐up interview)*
		‘Pacing myself is one of the biggest things and also knowing I don’t have to do it all in one go. I can let myself not do it because it’ll still be there tomorrow, whether it’s done or not*’ Diane (follow‐up interview)*

#### 3.7.1 Motivation and Attitude

Motivation to engage with the peer mentorship programme varied. Most mentees wanted to better understand and self‐manage their OA. However, for a couple of mentees, the programme functioned as a stopgap while awaiting surgery or physiotherapy.

Some mentees were highly motivated to self‐manage their OA pre‐intervention, and therefore readily engaged with the remote peer mentorship programme. In contrast, other mentees became motivated during the intervention. Mentees who perceived self‐management as preventative for OA progression were more motivated and engaged. Perceived improvements in OA symptoms, such as reduced pain and joint stiffness, served as motivating factors, leading to increased engagement with physical activity and the programme exercises.

Understanding how peer mentors managed their OA inspired some mentees to adopt similar strategies. Several mentees reflected on how the peer mentor or other mentee’s OA experiences had put their OA into perspective. However, one mentee, who observed that his peer mentor’s OA was more severe than his own, questioned the relevance of the recommended self‐management strategies for his situation.

Follow‐up interviews indicated that many mentees remained positive that they could self‐manage their OA. Ongoing motivation was driven by the programme, peer mentor, or due to increased mobility or reduced OA symptoms. A couple of mentees continued to self‐manage but had not fully accepted their life with OA and appeared to be grieving their former self.

### 3.8 Mediators

Engagement with self‐management was the most important factor that accounted for how or why outcomes occurred. All mediators are presented in Table 5.

**TABLE 5:** Intervention mediators.

Mediators	Description	Exemplar quotes
Demonstrating understanding of self‐management strategies	Findings from initial mentee interviews indicated that mentees were beginning to understand the purpose of self‐management strategies and how to practise them.	‘I expect it to get better every time. Because obviously the muscles are going to get stronger’ *Theresa (initial interview)*
		‘I’m so glad that, you know, I’ve received that help and I’ll have to continue, you know, like I said, it’s not something like if you have a headache and you take paracetamol and within half an hour it goes. It’s not like that, you know, you have to, you have to make effort, find the time and do the exercise’ *Anya (initial interview)*
Building confidence to use self‐management skills	Findings from initial mentee interviews demonstrated that most mentees were trialling a variety of self‐management strategies and finding out what worked best for their needs and developing the skills to perform the programme exercises.	‘That’s something I probably wouldn’t have specifically done. I’d have driven to McDonald’s or something with [granddaughter] or taken her to a cafe where we I could have parked. But I thought, no, we’ll park at the bottom, we’ll walk up. I ad her with me. So, you know, it wasn’t as if I was doing it on my own’ *Janice (initial interview)*
		‘Like before, it was very hard, difficult for me to bend my knee and now, now I hold the ledge and I bend properly my knees and things and then I do like a 10 or 12 of them in and I go slowly’ *Samira (initial interview)*
Knowledge of effective self‐management strategies	At follow‐up, mentees demonstrated a knowledge of self‐management strategies that they were confident would work, or were currently working for them.	‘I know that for my knee walking is going to be good. I keep up with it and also I found that because I’ve lost a little bit of weight it has made it a bit more easier to, you know, to bend down and put my socks on and stuff’ *Chika (follow‐up interview)*
		‘Because if you’re in pain and you know your knee won’t move, you’re frightened to do the exercises just in case it hurts it more. But it doesn’t, because it’s always going to be there. It’ll actually help it, because you’re moving it’ *Diane (follow‐up interview)*
Confidence to use self‐management strategies	Many mentees were confident that they were able to use some self‐management strategies that they believed were effective for managing their OA. For a small number of mentees, they were less confident to undertake certain activities.	‘I’m very confident I’ve got the tools that will help and build things back up again’ *Ernie (follow‐up interview)*
		‘When the problem started with the knee I couldn’t walk much but now I have started walking little bit more, not like more, more, because I’m always scared to go with this walking group’ *Kita (follow‐up interview)*
Engagement with self‐management strategies	Findings from initial mentee interviews showed that mentees were trying out self‐management including physical activity, programme exercises, healthy eating, pain management, relaxation techniques, goal setting and activity pacing. At follow‐up, some mentees reported a period of inactivity and a gradual return to self‐management, while other mentees spoke of regularly practising self‐management and appeared to have developed habits.	‘Simple things like if I’m going… like if I’m making a drink, you know I’ll do like the ones like for stretching my calves and things. I’ll do those while I’m waiting for the kettle to boil. You know, I’ll sit at the kitchen table and I’ll be standing up and sitting down, you know, without using the arms and things. It fits in during the day, and I’ll probably do more that way than what I would do if I did an exercise session, because it’s I’m, I’m moving around and I’m doing things as I’m moving’ *Denise (initial interview)*
		‘I had to get up and make an effort to get up and walk around the house or if I am in a little bit of pain, and sitting I use the sit down exercises… I Think the more exercises for the lower leg to move the ankle and move the knee joints’ *Olivia (follow‐up interview)*
		‘I did start doing it. I was really good for the first few weeks, and then, like I said, it just went out the window. I will hold my hand up. It’s my own fault’ *Janice (follow‐up interview)*
Support of others	Mentees discussed support they received from family, friends and practical support from joining walking groups.	‘I was going to the local shop with [partner’s] sister, I took the opportunity to walk to the shop without the aid of my walker […] I knew I had two people by me. I suddenly took that risk of going out without my walker’ *Olivia (follow‐up interview)*
		‘I wouldn’t miss the Monday morning walk ‘cause I wouldn’t go and do that on my own. Sometimes there might be three of us and sometimes there might be eight’ *Sheila (follow‐up interview)*
Reframing mindset	At follow‐up, some mentees discussed feeling like their mindset had changed from taking part in peer mentoring; with mentees reporting feeling more positive, less overwhelmed by their OA and being more accepting about having to do tasks differently.	‘I’ve taken away like positivity and you’ve got to adapt yourself. And also you gotta, not many, don’t get many negative thoughts in your mind as well, because you can try and you, if you try, you will do, get there eventually, you know, slowly and so don’t give up’ *Samira (follow‐up interview)*
		‘I know now that things are out of my control, and I can’t control everything like I thought I could, and by trying to think that I could control everything I was actually making the situation a lot worse. So now, by taking a big, deep breath and saying, I can’t change these things, let’s find another way around things. Yeah, so that’s definitely changed my mindset’ *Heather (follow‐up interview)*

#### 3.8.1 Engagement With Self‐Management

Initial interviews highlighted that most mentees were engaging with at least one self‐management strategy from the peer mentorship programme and had considered how to implement self‐management into their lives.

Data from follow‐up interviews indicated that if mentees were not implementing self‐management, this was typically due to a setback (e.g., illness), competing priorities, or because their OA pain was more manageable. For some, there was still a disconnect between understanding and implementing self‐management.

At follow‐up, most mentees were implementing activity pacing, while comparatively fewer mentees were actively setting goals. Some mentees continued programme exercises as a regular habit, alongside additional exercise. Other mentees had stopped or reduced how often they were practising programme exercises but were active via walking or exercise classes. More mentees reported activity at follow‐up compared with their baseline questionnaires.

## 4 Discussion

This process evaluation explored how suitable, acceptable, appropriate and useful participants found the remote peer mentorship programme, designed for people with hip and/or knee OA experiencing SED. The evaluation identified effective intervention components and highlighted elements that require further attention to optimise the intervention, such as fidelity to the programme content. Findings suggested that most mentees benefitted from the programme, despite the variation in delivery and inconsistent delivery of sessions that focussed on activity pacing and goal setting. The importance of a flexible and tailored programme to account for individual differences and preferences was highlighted. However, tailoring should be appropriately balanced with thorough programme delivery.

The role of the peer mentor within the peer mentorship programme was crucial for enhancing engagement. The programme was most successful when good rapport had been built and peer mentors shared their lived experience, were empathetic, listened and adapted sessions to suit the needs of their mentees. Findings from our previous in‐person peer mentorship support programme for people living with hip and/or knee OA emphasised the crucial role of the peer mentor, citing the mentee‐mentor connection as a significant enabler of self‐management through empowerment (Lavender et al. 2022b). Similar findings were evidenced by a scoping review of group‐based peer support programmes for adults with chronic illnesses (Stenberg et al. 2016) and a systematic review evaluating peer support interventions for adults with chronic pain (Arnott et al. 2023), which highlighted the importance of the unique peer‐to‐peer relationship and reported support, shared experience/understanding, communication, personal connection and not feeling alone as beneficial components of peer mentorship that encourage self‐management.

Perceiving the peer mentorship programme as useful, and gaining something from taking part appeared to play a key role in mentees’ engagement with the programme and self‐management. Mentees cited increased knowledge of self‐management strategies and improved confidence to self‐manage their OA as the primary gains from engaging with peer mentorship. Programme benefits that mentees reported included: ability to do more activity, losing weight, improved mobility, decrease in OA pain/symptoms and feeling less overwhelmed and more able to self‐manage their condition. This aligns with findings from a scoping review that identified learning new ways of perceiving situations and obtaining knowledge and skills as key factors that can lead to behaviour changes (Stenberg et al. 2016).

Engagement with self‐management and practising strategies introduced during the peer mentorship programme appeared to be related to mentee achievements around physical activity, eating better, and improved OA symptoms. Qualitative data indicated that more mentees were undertaking physical activity compared with figures reported in their baseline data. However, findings also suggest that practising exercise is complex and impacted by a variety of contextual factors, especially managing other long‐term health conditions and competing priorities such as caring responsibilities and work commitments. Evidence from a systematic review and qualitative synthesis of 26 studies focussed on self‐management of diabetes found that people with multiple long‐term conditions and experiencing SED often faced competing priorities and time constraints, making it difficult to self‐manage their diabetes alongside work or caregiving, with others’ needs frequently taking precedence (Woodward et al. 2024).

The remote intervention format was generally acceptable, but in some instances, it slowed the development of rapport. To address barriers that people experiencing SED may encounter, relating to digital awareness, literacy, and access, mentees were supported to use videoconferencing. Remote programme delivery was less challenging than anticipated. Mentees were also given the option of telephone‐based peer mentorship, which allowed flexibility for mentees to participate in their preferred remote format and follow recommendations to offer alternative, non‐digital options (Arthritis and Musculoskeletal Alliance 2024). Some mentees highlighted the convenience of remote peer mentorship, that aligned better with their schedules than traditional healthcare. Other mentees also valued the consistent, dedicated, unrushed time with their peer mentor, that they viewed as more useful than appointments with healthcare professionals. These views suggest that the peer mentorship programme helped to address some of the barriers faced by people with OA who are experiencing SED identified in our previous work, such as inadequate support from healthcare professionals (Anderson et al. 2025). These insights also emphasise that person‐centred care for people living with SED must fit around the competing priorities in their lives and provide them with adequate time.

Overall, the remote peer mentorship programme was well received and perceived as acceptable, enjoyable and useful. Acceptability of the programme influenced all aspects of the process evaluation and shaped programme engagement. However, programme acceptability did not necessarily translate into sustained programme adherence. While findings indicate that contextual factors can shape behaviour more significantly than acceptability, additional issues were identified. One mentee dropped out due to a lack of peer mentor availability, and another struggled to connect remotely. Given that some mentees withdrew from the programme alongside variable attendance and engagement, it is likely that elements of the programme were not acceptable.

### 4.1 Limitations

Programme attendance was variable. Mentees faced a variety of competing demands, which made it challenging to consistently prioritise the programme. On reflection, several mentees stated that the timing of the sessions had been inconvenient. This emphasises the need for dynamic scheduling, greater flexibility and responsiveness in programme delivery to accommodate the variety of complex needs presented by mentees.

The remote peer‐mentorship format may have been a barrier to recruitment, with people who were more comfortable and confident digitally self‐selecting to take part in the programme. As such, findings are less likely to be applicable to those experiencing SED who experience digital exclusion.

A further limitation was that the evaluation could not demonstrate how the remote peer mentorship programme overcame certain barriers that people who experience SED may have, as the interview topic guide didn’t include questions about whether mentees had experienced barriers to accessing traditional healthcare. Mentee interview data mainly explored the convenience and usefulness of the remote format.

Generalisability of the findings is also limited beyond the cohort of SED participants living with hip and/or knee OA in England. Replication of this study in other countries would likely require methodological adaptations to account for differences in healthcare systems, care pathways and guidelines, and to reflect where research and service provision gaps exist. Differences between countries in health behaviours and broader health and social inequalities, including disparities in healthcare access, digital inclusion, and resource utilisation, may further impact comparability.

### 4.2 Implications for Practice and Future Research

Three key challenges to implementing a remote OA peer mentorship programme for people experiencing SED were identified.

The first is producing strategies that support the development of peer mentor‐mentee rapport, as the relationship between the peer mentor and mentee was key to programme engagement, and is supported by literature (Stenberg et al. 2016; Lavender et al. 2022a, 2022b; Arnott et al. 2023). When matching a mentee to a peer mentor, programme co‐ordinators should consider matching based on commonalities other than OA and consider re‐matching the mentee with another peer mentor if rapport is challenging. Findings from our previous in‐person peer mentorship programme noted that accounting for personality traits during the matching process facilitated the development of the mentor‐mentee relationship and impacted mentee accountability (Lavender et al. 2022a).

The second challenge is successfully encouraging mentees with complex lives and competing priorities to prioritise self‐managing their OA. Strategies could include: offering a buddy system, where mutual support is emphasised over traditional mentor‐mentee dyad; offering flexibility around programme structure and timing; and delivering mentorship support alongside other health services.

The third challenge is to address the support needs of the peer mentor so that they can appropriately and confidently deliver the peer mentorship programme. These needs could potentially be met by offering refresher training sessions to ensure that peer mentors feel knowledgeable and able to develop and refine their skills.

## 5 Conclusion

This study demonstrated that remote peer mentorship for people with OA experiencing SED is largely acceptable, with the potential for meaningful impact. Findings suggested that the approach helped to improve OA self‐management among people living with hip and/or knee OA and experiencing SED. To optimise future delivery, investment is needed in mentee recruitment and scheduling strategies, peer mentor training and support, support with digital capabilities and flexible approaches that account for the complexity of peoples’ lives.

## Author Contributions

**Samantha Mason:** data collection and curation, analysis, writing original draft, review, editing and final approval. **Anna M. Anderson:** conceptualisation, funding acquisition, methodology, analysis, review, editing and final approval. **Elizabeth C. Lavender:** conceptualisation, funding acquisition, methodology, data collection and curation, analysis, review, editing and final approval. **Linda Eckersley:** conceptualisation, funding acquisition, methodology, review, editing and final approval. **Susan Barry:** conceptualisation, funding acquisition, methodology, review, editing and final approval. **Mark Conner:** conceptualisation, funding acquisition, methodology, review, editing and final approval. **Heidi R. Green:** conceptualisation, funding acquisition, methodology, review, editing and final approval. **Gretl A. McHugh:** conceptualisation, funding acquisition, methodology, data collection and curation, analysis, review, editing and final approval.

## Funding

This project was funded by the Nuffield Foundation’s Oliver Bird Fund and Arthritis UK (grant number: OBF/FR‐000023819), but the views expressed are those of the authors and not necessarily the funders. Visit www.nuffieldfoundation.org and www.arthritis‐uk.org.

## Conflicts of Interest

The authors declare no conflicts of interest.

## Supporting information


Supporting Information S1

## Data Availability

The data that support the findings of this study are available on request from the corresponding author. The data are not publicly available due to privacy or ethical restrictions.
