# Supplementary material for: Evaluating the Delivery of a Remote Osteoarthritis Peer Mentorship Programme for People Experiencing Socioeconomic Disadvantage
Source: Musculoskeletal Care. 2026 Nov 6;24(4):e70297. doi: 10.1002/msc.70297 (PMC13743082; doi:10.1002/msc.70297)
Supplement: Supplementary file 1 [file MSC-24-e70297-s001.docx]

**Supplementary files**

**Supplementary table 1: Consolidated criteria for reporting qualitative studies (COREQ): 32-item checklist**

| **No. Item** | **Guide questions/description** | **Reported on Page #** |
| --- | --- | --- |
| **Domain 1: Research team and reﬂexivity** |  |  |
| *Personal Characteristics* |  |  |
| 1. Interviewer/facilitator | Which author/s conducted the interview or focus group? | 9 |
| 2. Credentials | What were the researcher’s credentials? E.g. PhD, MD | Available via ORCID profile |
| 3. Occupation | What was their occupation at the time of the study? | 10 |
| 4. Gender | Was the researcher male or female? | 10 |
| 5. Experience and training | What experience or training did the researcher have? | 10 |
| *Relationship with participants* |  |  |
| 6. Relationship established | Was a relationship established prior to study commencement? | Not reported |
| 7. Participant knowledge of the interviewer | What did the participants know about the researcher? e.g. personal goals, reasons for doing the research | Not reported |
| 8. Interviewer characteristics | What characteristics were reported about the inter viewer/facilitator? e.g. Bias, assumptions, reasons and interests in the research topic | Not reported |

| **Domain 2: study design** |  |  |
| --- | --- | --- |
| *Theoretical framework* |  |  |
| 9. Methodological orientation and Theory | What methodological orientation was stated to underpin the study? e.g. grounded theory, discourse analysis, ethnography, phenomenology, content analysis | Abstract, 4, 5. |
| *Participant selection* |  |  |
| 10. Sampling | How were participants selected? e.g. purposive, convenience, consecutive, snowball | 5-6, figure 1 |
| 11. Method of approach | How were participants approached? e.g. face-to-face, telephone, mail, email | 5-6 |
| 12. Sample size | How many participants were in the study? | 5-6, 12-13 figure 1 |
| 13. Non-participation | How many people refused to participate or dropped out? Reasons? | Figure 1, Supplementary table 2 |
| *Setting* |  |  |
| 14. Setting of data collection | Where was the data collected? e.g. home, clinic, workplace | 8 |
| 15. Presence of non-participants | Was anyone else present besides the participants and researchers? | N/A |
| 16. Description of sample | What are the important characteristics of the sample? e.g. demographic data, date | 12, table 1 |
| *Data collection* |  |  |
| 17. Interview guide | Were questions, prompts, guides provided by the authors? Was it pilot tested? | 8-9 |
| 18. Repeat interviews | Were repeat inter views carried out? If yes, how many? | 13 |
| 19. Audio/visual recording | Did the research use audio or visual recording to collect the data? | 9 |
| 20. Field notes | Were ﬁeld notes made during and/or after the interview or focus group? | 9 |
| 21. Duration | What was the duration of the interviews or focus group? | 9 |
| 22. Data saturation | Was data saturation discussed? | N/A as all mentees and peer mentors were invited to participate in qualitative interviews and using the concept of saturation is advised against in studies employing reflexive thematic analysis |
| 23. Transcripts returned | Were transcripts returned to participants for comment and/or correction? | Not reported |
| **Domain 3: analysis and ﬁndings** |  |  |
| *Data analysis* |  |  |
| 24. Number of data coders | How many data coders coded the data? | 10 |
| 25. Description of the coding tree | Did authors provide a description of the coding tree? | Not reported |
| 26. Derivation of themes | Were themes identiﬁed in advance or derived from the data? | 10 |
| 27. Software | What software, if applicable, was used to manage the data? | 10 |
| 28. Participant checking | Did participants provide feedback on the ﬁndings? | Not reported |
| *Reporting* |  |  |
| 29. Quotations presented | Were participant quotations presented to illustrate the themes/ﬁndings? Was each quotation identiﬁed? e.g. participant number | Yes; tables 3 – 5, pages 14-18. |
| 30. Data and ﬁndings consistent | Was there consistency between the data presented and the ﬁndings? | Yes; pages 12-22, tables 1 – 5. |
| 31. Clarity of major themes | Were major themes clearly presented in the ﬁndings? | Yes; pages 13 - 17 |
| 32. Clarity of minor themes | Is there a description of diverse cases or discussion of minor themes? | Not reported/not applicable |

**Supplementary table 2: Remote peer mentoring sessions - details and structure**

| **Topic name** | **Rationale for topic inclusion** | **Suggested structure for presenting topic** |
| --- | --- | --- |
| Core topic 1: Self-managing OA | Providing an understanding of osteoarthritis, and knowledge and understanding of a number of self-management tools, to help their mentee to manage their symptoms better | • Suggested to be introduced during week one or two • Explore mentee's views of osteoarthritis by discussing facts and myths around the condition • Explain what self-management tools are, introduce the tools covered in future sessions and explain that these tools are based on research and clinical experience  • Understand how the mentee currently manages their symptoms  • Discuss any self-management tools the mentee would like to know more about • Refer mentee to Arthritis UK osteoarthritis booklet for more information |
| Core topic 2: Setting realistic goals | Providing support to mentee to set and review simple and meaningful weekly goals | • Suggested to be introduced during week one or two • Discuss key points about goal setting (why set a goal, what goal setting is, how to set goals and SMART* goals) • Ask mentee to review goals at each subsequent support session • Where goals have not been met, discuss problem-solving tips and ways to address and manage this  • Encourage adaptation of goals if they are too ambitious |
| Core topic 3: Getting active, staying active | Being physically active is key to self-management of osteoarthritis and also helps to improve overall health and wellbeing | • Suggested to be introduced during week one or two • Discuss the getting active, staying active handout. This handout explains how and why being physically active is one of the best ways to manage osteoarthritis, health and wellbeing benefits of being active, how to build an active lifestyle, and reinforces the importance of setting a physical activity goal • Introduce home activity plan of exercises and encourage mentee to try the plan. Peer mentor is encouraged to do some exercises with mentee • Encourage mentee to consider what physical activities they enjoy or would like to try • Discuss how to break up periods of rest and sitting  • Support mentee to set at least one physical activity goal and review this during subsequent sessions • If appropriate, discuss preventing falls with mentee |
| Core topic 4: Eating well, feeling well | Healthy eating has multiple benefits, including helping osteoarthritis pain through weight management | • Suggested to be introduced during week two or three • Suggested to begin by explaining to the mentee that it is their choice to discuss this topic, as healthy eating and weight management can be sensitive and there may be multiple reasons why the mentee may find it hard to follow a specific diet  • Support mentee to set healthy eating and/or weight loss goals if they wish • Work through the eating well, feeling well handouts, as appropriate. The handouts explain what eating healthily means, how to eat healthily on a budget, what else can be done to manage weight, how to set healthy eating or weight loss goals and includes an eat well plate (visual tool for a healthy, balanced diet) |
| Core topic 5: Activity pacing in practice | Activity pacing can help to reduce the 'boom-bust cycle' - where people over-do activities on days where their symptoms are less severe, leading to a flare-up of their symptoms and needing a period of rest | • Suggested to be introduced during week two or three • Discuss the reasons to use activity pacing  • Use the boom-bust cycle to explain patterns of overdoing things followed by periods of rest or inactivity and how this can reduce a person's ability to stay active, which can impact physical and mental wellbeing **•** Explain how activity pacing can prevent boom-bust cycling, achieve more consistent activity levels and reduce symptom flare-ups • Refer to the activity pacing section of the resources. This resource helps to understand how the mentee currently carries out activities and whether they currently experience boom-bust, how activities affect their symptoms, what a baseline level of activity looks like, adjusting or adapting activities to make them more manageable, implementing consistent activity and a flexible approach to activity pacing, knowing when to stop certain activities and carry on with others, and building activity over time • Use the activity pacing in practice handout to help the mentee build activity pacing into daily life |
| Core topic 6: Managing pain | Pain is usually one of the main symptoms of osteoarthritis. Self-management strategies and tools to help manage pain better can positively affect the mentee | • Suggested to be introduced during week two or three • Introduce self-management strategies and tools that the mentee has not engaged with in-depth, especially if they are also working on getting active and activity pacing • Refer to the pain and osteoarthritis section of the resource booklet. This explains why osteoarthritis causes pain, what the persistent pain cycle is, its physical and mental impact, and the best ways to manage pain • Use the managing pain handout to support discussions with the mentee |
| Optional topic 1: Getting motivated | Some mentees may struggle to find the motivation to try self-management and may need support and help to find solutions | • Optional topics can be covered with the mentee at any point during the peer mentorship programme  • Review the supporting your participant's emotional wellbeing section of the resource booklet. This instructs peer mentors not to tell mentees to be more positive, but to empathise with them about challenges they are facing and, if appropriate, share how the peer mentor has overcome setbacks • Use the getting motivated handout to discuss low mood and how they want to get back on track if the mentee feels demotivated |
| Optional topic 2: Relaxing and sleeping | Sleeping and relaxing can be challenging when living with OA. There are a range of techniques that may help a mentee to relax and sleep better | • Optional topics can be covered with the mentee at any point during the peer mentorship programme • This topic may be especially useful to cover if the mentee mentions that they often feel stressed and anxious, or that they have problems relaxing and sleeping • Refer to the why focus on relaxation and sleep section of the resource booklet • Review the relaxing and sleeping handout with the mentee. The handout includes three relaxation techniques and suggestions for how to sleep better • Refer to the using relaxation techniques and getting a better sleep sections of the resource booklet for guidance on how to support the mentee to try relaxation techniques and sleep suggestions, which include having a regular sleep routine, minimising daytime sleeping, exercise, getting natural daylight outside and addressing diet, caffeine and alcohol intake |
| Optional topic 3: Communicating well | Communicating better can help relationships with family, friends and health professionals. Help to communicate assertively with healthcare professionals can help the mentee to better advocate for themselves | • Optional topics can be covered with mentee at any point during the peer mentorship programme • This topic may be useful to cover if the mentee indicates that they are having issues communicating with healthcare professionals • Review the communicating well handout to explore communication style(s)  • If the mentee has problems communicating assertively, talk through tips for assertive communication • Suggest that the mentee keeps a diary to documents symptom changes and general ups and downs to help with clear communication and remembering details when discussing their health with healthcare professionals |
| Optional topic 4: Getting connected | Encouraging the mentee to make other connections will help their wellbeing and plan ahead for when the peer mentorship ends. | • Optional topics can be covered with mentee at any point during the peer mentorship programme • If this topic hasn't been discussed by the fifth session, but it would help the mentee, raise it at this stage • Some mentees may find it challenging to connect with services, groups and activities, or keep in touch with family or friends • Use the getting connected handout to discuss services, groups, and activities that the mentee may want to be involved with. If the mentee wants to join a service, group or undertake an activity, encourage this before the final mentoring session, so you can discuss how it went • Suggest taking a friend or family member for support if the mentee is reluctant • If the mentee finds leaving the house challenging, a brief outing may be a key first step |
| Optional topic 5: Having a joint replaced | If the mentee is waiting for a joint replacement, the self-management tools could help them to prepare for the operation and for recovery afterwards | • Optional topics can be covered with mentee at any point during the peer mentorship programme • The mentee may find this topic useful if they're waiting for a joint replacement or are considering having a joint replaced • Discuss what a joint replacement is using the handout • Discuss how they could prepare and what to expect at each stage of the joint replacement journey • If the peer mentor has had a joint replaced, they may wish to share their experience • Option to have a member of the PPIE group involved in this session, who had experience of joint replacement |
| Optional topic 6: Getting support for work | There are lots of support options available that may help if the mentee's OA symptoms are affecting their ability to work | • Optional topics can be covered with mentee at any point during the peer mentorship programme • This topic may be relevant if the mentee is currently working, on sick leave or looking for work • Use the handout to talk about what support options may be helpful to the mentee • The mentee may find activity pacing useful at work |

*SMART goals - Specific, Measurable, Achievable (or Attainable), Relevant, and Time-Bound

**Supplementary table 3: Reasons for mentee withdrawal or non-completion of remote peer mentorship sessions**

| **Mentee** | **Withdrawal or non-completion of sessions** | **No of sessions completed** | **Reason for withdrawal** |
| --- | --- | --- | --- |
| 1 | Active withdrawal by mentee | 3 | Inconvenient timing of peer mentorship sessions |
| 2 |  | 2 | Inconvenient timing of peer mentorship sessions |
| 3 |  | 2 | Inconvenient timing of peer mentorship sessions |
| 4 |  | 1 | Family or personal reasons |
| 5 |  | 1 | Family or personal reasons |
| 6 |  | 3 | Lack of peer mentor availability |
| 7 |  | 3 | Felt she got what she needed from the programme |
| 8 | Passive withdrawal by mentee, then withdrawn by study team | 2 | Non-attendance of peer mentorship sessions, mentee could not be contacted |
| 9 |  | 2 | Non-attendance of peer mentorship sessions, mentee could not be contacted |
| 10 |  | 1 | Non-attendance of peer mentorship sessions, mentee could not be contacted |
| 11 |  | 3 | Peer mentor informed study team that mentee had family and personal circumstances that had come up and had sustained an injury, unrelated to the programme, mentee could not be contacted |
| 12 | Non-completion of one or more sessions | 5 | Missed one session due to illness |
| 13 |  | 5 | Considered themselves to have completed the programme after five sessions |
| 14 |  | 5 | Considered themselves to have completed the programme after five sessions |
| 15 |  | 5 | Considered themselves to have completed the programme after five sessions |
| 16 |  | 4 | Missed two sessions due to illness and family caring responsibilities |
| 17 |  | 3 | Inconsistent attendance, no reason provided and no withdrawal from study |
| **Total: 17** | | | |
